# Modelling, Analysis and Validation of Hydraulic Self-Adaptive Bearings for Elevated Floating Bridges

**DOI:** 10.3390/s24248079

**Published:** 2024-12-18

**Authors:** Lianpeng Zhang, Yuan Liu, Tailai Yang, Ruichen Wang, Jie Feng, David Crosbee

**Affiliations:** 1 School of Mechanical Engineering, Shijiazhuang Tiedao University, Shijiazhuang 050043, China; zhanglianpeng@stdu.edu.cn (L.Z.); ly1383221105@163.com (Y.L.); yangtailai2024@163.com (T.Y.); fengjie@stdu.edu.cn (J.F.); 2 Institute of Railway Research, University of Huddersfield, Huddersfield HD1 3DH, UK; d.crosbee@hud.ac.uk

**Keywords:** hydraulic self-adaptive bearing system, flexible support control, vibration control, multi-state observer control, three-variable control

## Abstract

Conventional floating bridge systems used during emergency repairs, such as during wartime or after natural disasters, typically rely on passive rubber bearings or semi-active control systems. These methods often limit traffic speed, stability, and safety under dynamic conditions, including varying vehicle loads and fluctuating water levels. To address these challenges, this study proposes a novel Hydraulic Self-Adaptive Bearing System (HABS). The system integrates real-time position closed-loop control and a flexible support compensation method to enhance stability and adaptability to environmental changes. A modified three-variable controller is introduced to optimise load response, while a multi-state observer control strategy effectively reduces vibrations and improves traffic smoothness. A 1:15 scale prototype was constructed, and a co-simulation model combining MATLAB/Simulink and MSC Adams was developed to simulate various operational conditions. Results from both experiments and simulations demonstrate the HABS’s ability to adapt to varying loads and environmental disturbances, achieving a 72% reduction in displacement and a 54% reduction in acceleration. These improvements enhance traffic speed, stability, and safety, making the system a promising solution for emergency and floating bridges, providing superior performance under challenging and dynamic conditions.

## 1. Introduction

Railway transportation is a vital mode of passenger and cargo transport, contributing significantly to global economic growth. With over 200,000 railway bridges in China, including 30,000 for high-speed rail, these structures face increasing challenges from heavy traffic loads and changing environmental conditions. Elevated floating bridges, essential for emergency repairs during floods or earthquakes, are particularly complex and costly. Conventional rubber bearings used in these repairs struggle to adapt to fluctuating loads and water levels, compromising stability and safety. Advanced compensation techniques are urgently needed to improve the performance and reliability of elevated floating bridge systems under demanding conditions.

Wave heave compensation technology, initially developed in the 1960s, was first used in semi-submersible drilling ships and marine equipment. Over time, its applications expanded to include submersible retraction, cargo transfer, offshore replenishment, and ocean mining, making it a critical technology in marine industries [1,2]. Li [3] introduced a hybrid active–passive system with a nonlinear cascade controller and an adaptive disturbance observer, significantly improving dynamic response and operational efficiency. K.D. [4] developed an active system with an electro-hydraulic actuator to stabilise riser movements during offshore drilling. Neupert [5] proposed a predictive control strategy to decouple payload dynamics from vessel motion, enhancing precision in tasks like offshore construction. Additionally, a crown-block heave compensation system was designed using compound cylinders and sliding mode control to improve performance in severe wave conditions [6]. Li [7] developed a double-loop controller for hydraulic valve-based active heave compensation, combining disturbance rejection and model predictive control for better stability and adaptability. Sanchez [8,9] addressed seal friction in hydro-pneumatic passive heave compensators, creating a control system that reduced heave motion and vibration, outperforming conventional feedforward controllers. Yu [10] introduced variable structure control to active heave systems, improving robustness and operational stability in dynamic offshore conditions.

Hydraulic control systems have become a focal point in recent research due to their versatility and effectiveness in various industries. Zhang developed a semi-automatic system for rotary forging, improving deformation uniformity and material efficiency [11]. Wang used electro-hydraulic controls to enhance stability and safety in mining and tunnelling [12]. Liu addressed internal leakage in turbine inlet valves, creating a mathematical model to reduce energy loss [13], while Ni studied servo valve failures in critical systems, improving reliability in power generation and aerospace [14]. Yang tackled cavitation in aircraft hydraulic systems, extending component lifespan with innovative nozzle designs [15]. Zhang explored fluid transmission in press machines, improving efficiency and reliability [16]. An developed a hydraulic-pneumatic system for rail grinding cars, focusing on precision control to extend track life [17]. Kong optimised fault diagnosis with a sensor placement method, reducing diagnostic times and boosting reliability [18]. Ma designed an adaptive PID controller for excavators, enhancing performance under varied conditions [19]. Guo created a servo electro-hydraulic system for heavy extruders, prioritising energy efficiency and sustainability [20].

Due to the fundamental challenges associated with the low resonance frequency and small damping ratio of electro-hydraulic servo systems, the three-variable control (TVC) method addresses the challenges of low resonance frequency and small damping ratios in electro-hydraulic servo systems. By combining feedforward and feedback control with acceleration and velocity feedback, TVC improves frequency bandwidth and system stability, enhancing precision in hydraulic shaking tables [21,22]. Yang [23] optimised TVC parameters, using the root locus method for finer control, while Shen [24] simplified parameter tuning with a design based on acceleration and displacement signals. Yang [25] demonstrated TVC’s effectiveness in continuous swept-sine vibration control, and Zhang [26] advanced its application by improving the frequency response of hydraulic shaking tables, accounting for structural flexibility.

Floating bridge bearing systems are highly affected by time-varying disturbance forces, requiring advanced control strategies for effective mitigation. Lin [27] developed a neural state observer for managing disturbances in spacecraft attitude control. Xu [28] used integral sliding mode control to eliminate disturbances in motor systems, while Ghadiri [29] applied a nonlinear sliding mode controller to reduce external forces in gimbal systems. Nguyen [30] introduced a neural network-based adaptive robust control for handling uncertainties in electro-hydraulic servo systems. Benevides [31] designed a disturbance observer combining robust linear quadratic regulation and a Kalman filter to improve trajectory tracking in quadrotors. Yogi [32] proposed an adaptive sliding mode control using a neural network to ensure fast convergence and improved disturbance handling in quadrotor systems.

To achieve effective vibration rejection control in lightweight, flexible single-link arms, Wang [33] proposed a robust control method combining PD control, ADRC, and sliding mode compensation to achieve precise vibration rejection in lightweight single-link arms. Chen [34] enhanced ADRC with fast terminal-sliding mode control for improved tracking in lower limb exoskeletons. For induction motors, an ADRC approach with adaptive particle swarm optimisation improved decoupling and disturbance compensation [35]. Fareh [36] introduced a robust ADRC for flexible manipulators, enabling accurate trajectory tracking and vibration suppression. Feliu [37] developed a robust control strategy for trajectory tracking in lightweight flexible arms, minimising vibrations. Morales [38] proposed a two-nested loop robust control for flexible arms, effectively handling structural flexibility. Pereira [39] employed integral resonant control with nested feedback loops for flexible manipulators, while Sayahkarajy [40] combined MIMO H∞ feedback control with a pre-shaping feedforward controller to suppress vibrations in two-link elastic manipulators.

Further advancements were conducted: Jiang [41] tackled unknown spatio-temporal disturbances in flexible beam systems using a disturbance observer and neural network control with full-state and output feedback, achieving vibration suppression and handling dynamic uncertainties. Copot [42] developed an intelligent disturbance filter to improve vibration control in complex mechanical multi-body systems. Liu [43] applied a hybrid backstepping-boundary iterative learning control method to manage vibrations in Euler-Bernoulli beam systems under external disturbances. Jiang [44] later combined modal characteristics, differential flatness, and active disturbance rejection control to stabilise long flexible arms, enhancing both stability and vibration resistance. Building on these advancements, this study introduces a Hydraulic Self-Adaptive Bearing System (HABS) to address limitations in traditional floating bridge systems, such as reduced traffic speed and stability. The HABS features an electro-hydraulic control system with servo valves managing hydraulic cylinders for position closed-loop control, minimising bridge displacement under varying loads. A modified three-variable controller improves overall performance, while a multi-state observer control strategy with a PD controller reduces vibrations, ensuring enhanced stability and reliability in dynamic environments.

To address the limitations in traffic speed, stability, and safety posed by traditional floating bridge systems, this paper introduces a novel Hydraulic Self-Adaptive Bearing System (HABS). Through the servo control system, position closed-loop control is achieved, allowing the system to maintain minimal bridge displacement even under significant variations in load conditions. The system model further incorporates the flexibility of the bridge’s support structure, with the introduction of a modified three-variable controller aimed at enhancing overall performance. Additionally, to reduce bridge vibrations caused by dynamic loads and environmental factors, a multi-state observer control strategy, based on a Proportional-Derivative (PD) controller, is developed.

A test-rig was established using a 1:15 scale model of the bridge, facilitated by xPC Target rapid prototyping technology, providing a platform for real-time testing and system validation. Parallel to the test-rig setup, a co-simulation model was developed using MATLAB/Simulink and MSC Adams software, simulating the dynamic behaviour and operational conditions of the bridge system. Both predictive and experimental analysis are conducted to evaluate the feasibility and effectiveness of the proposed HABS design and control strategies. In addition to addressing the limitations of conventional floating bridge systems, the HABS offers a flexible framework adaptable to various dynamic applications. Its modular design ensures suitability for emergency repairs and various dynamic applications, while its scalability allows for its broader adoption in modular and long-term infrastructure projects.

This paper is organised into six sections. Section 2 presents the proposed HABS concept, particularly its application in emergency bridge repair scenarios. Section 3 outlines the position closed-loop control architecture, detailing the design of the modified three-variable controller and the PD-based multi-state observer control strategy, which together enable adaptation-to-load fluctuations and water level changes while mitigating structural vibrations. Section 4 describes the development and implementation of the co-simulation model for the scaled bridge system, followed by a discussion of the simulation results. Section 5 elaborates on the validation, including the setup and findings from real-time tests. Finally, Section 6 provides a comprehensive conclusion, summarising the key findings and contributions of this research.

## 2. The Proposed Hydraulic Self-Adaptive Bearing System (HABS)

The novel Hydraulic Self-Adaptive Bearing System (HABS) is introduced to address the limitations of conventional floating bridge systems, which often struggle with stability and adaptability under varying load conditions. The proposed system utilises a servo-controlled hydraulic mechanism designed to accommodate real-time adjustments in response to dynamic vehicle loads and fluctuating environmental factors, such as water levels. By incorporating advanced control strategies, the HABS enhances bridge stability and safety, even in challenging operational environments.

### 2.1. Overview of HABS Design

**Figure 1:**
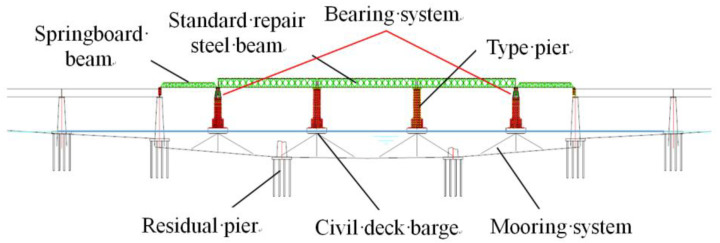
Emergency repair scheme of elevated deep water floating bridge.

Figure 1 presents a widely employed emergency repair scheme for elevated deep-water railway bridges. The structural complexity of these floating bridges, combined with their relatively low rigidity, often results in significant vibrations when trains traverse the bridge. The conventional rubber bearing systems typically used in such applications impose considerable constraints on traffic speed, operational stability, and overall safety, particularly under conditions of fluctuating water levels and dynamic loads. As railway infrastructure meets increasing demands, traditional bearing systems are increasingly inadequate for modern performance requirements. To address these challenges, the present study introduces a novel Hydraulic Self-Adaptive Bearing System (HABS), featuring an advanced electro-hydraulic servo control mechanism. The proposed HABS incorporates real-time position closed-loop control, enabling continuous adaptation to changing conditions. Additionally, the system integrates a multi-state observer control strategy based on a Proportional-Derivative (PD) controller, aimed at effectively mitigating bridge vibrations and compensating for displacements resulting from variations in vehicle load and water level. This adaptive control approach ensures enhanced stability, safety, and operational reliability, making it a suitable solution for modern elevated railway bridge applications under dynamic and fluctuating conditions.

**Figure 2:**
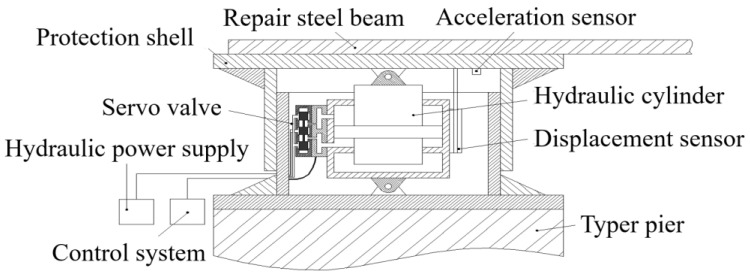
The proposed novel HABS structure.

**Figure 3:**
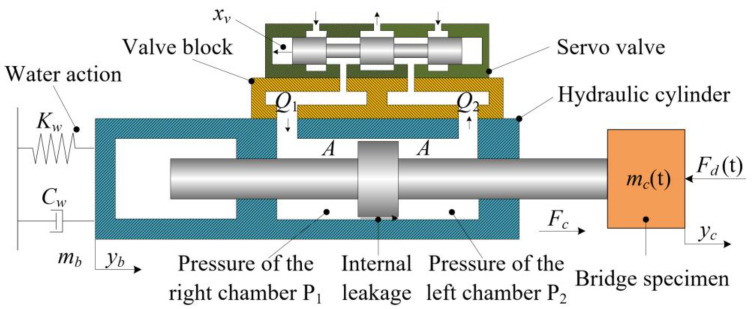
The simplified working principle of the HABS.

Figure 2 presents the proposed Hydraulic Self-Adaptive Bearing System (HABS), which integrates several key components critical for its operation. These components include an electro-hydraulic servo valve, a dynamic hydraulic cylinder, a valve block, a hydraulic power supply unit, a servo motion controller, displacement and acceleration sensors, and a protective metal casing. Each of these elements plays a critical role in ensuring the system’s efficiency and responsiveness. Figure 3 illustrates the fundamental working principle of the HABS. In this configuration, the flexible bridge specimen is conceptualised as a time-varying mass load, which is subjected to external disturbances that change over time. The interaction between the bridge system and the water environment is represented by a spring-mass-damper model, which accounts for the complex hydro-elastic effects that influence the stability of the structure.

The HABS utilises a real-time position closed-loop control mechanism, specifically engineered to minimise displacement at the bridge ends and mitigate acceleration responses across the entire bridge structure. This control approach is designed to adapt continuously to dynamic changes, ensuring the stability of the bridge even under variable conditions such as fluctuating water levels and shifting vehicle loads. The reference input signal for the control system is maintained at zero, allowing the system to continuously regulate itself without manual intervention. To achieve this purpose, the displacement and acceleration data from various critical points on the bridge, such as the ends, midspan, and floating pier, are continuously collected by the displacement and acceleration sensors. The detected signals are processed by the servo motion controller, which then adjusts the hydraulic flow and the output force of the hydraulic cylinder accordingly. By dynamically adapting to the changing conditions, including vehicle loading and water disturbances, the HABS optimises the structural performance and stability of the bridge, ensuring safe and efficient operations in real-world conditions.

### 2.2. Dynamic Modelling of HABS

As shown in Figure 2 and Figure 3, the Hydraulic Self-Adaptive Bearing System (HABS) can be modelled as a hydraulic cylinder system regulated by a servo valve. The configuration accommodates a flexible bridge specimen subjected to dynamic vehicle loads, which rests on a flexible foundation experiencing displacement variations. Precise control is required to ensure stability and maintain system performance. The mathematical model performing the behaviour of the servo valve, responsible for regulating fluid flow and pressure within the hydraulic cylinder, is expressed as follows:

(1) $$G_{sv}(s)=\frac{x_{v}}{i_{A}}=\frac{K_{v}}{\left(\frac{s^{2}}{w_{v}^{2}}+\frac{2\xi_{v}}{w_{v}}s+1\right)}$$

where *x_v_* is the displacement of the servo valve, *i_A_* is the servo valve drive current, *K_v_* is the servo valve power amplifier gain, *w_v_* is the natural angular frequency of the servo valve, and *ξ_v_* is the damping ratio of the servo valve.

The linearised load flow from the servo valve to the hydraulic cylinder, while disregarding the effects of hydraulic cylinder leakage and hydraulic oil compression, is represented by the following equation:

(2) $$Q_{L}=K_{q}x_{v}-K_{c}P_{L}$$

where *P_L_* is the load pressure, defined by *P_L_ = P_1_−P_2_*, in which *P_1_* and *P_2_* are the pressures inside the two chambers of the cylinder. And the average load flow can be obtained as *Q_L_* = *Q_1_* + *Q_2_*/2, in which *Q_1_* and *Q_2_* are the flow in and out of the servo valve, *K_c_* is defined as the flow pressure coefficient and *K_q_* is a linearised flow gain coefficient, and can be expressed as follows:

(3) $$K_{q}=\frac{\partial Q_{L}}{\partial x_{v}}=C_{d}w\sqrt{\frac{P_{S}-P_{L}}{\rho }}$$

(4) $$K_{c}=\frac{\partial Q_{L}}{\partial P_{L}}=\frac{C_{d}wx_{v}}{2}\sqrt{\frac{1}{\rho \left(P_{S}-P_{L}\right)}}$$

where *C_d_* is the discharge coefficient, *w* is the constant area gradient of the servo valve orifices, *ρ* is the mass density of the fluid, and *P_S_* is the hydraulic supply pressure, defined by *P_s_ = P_1_ + P_2_*. With consideration of the total leakage coefficient, *Q_L_* can be obtained by the flow continuity equation from the servo valve to the hydraulic actuator, given as follows:

(5) $$Q_{L}=A_{p}\frac{d\left(y_{c}-y_{b}\right)}{dt}+C_{tp}P_{L}+\frac{V_{t}}{4\beta_{e}}\frac{dP_{L}}{dt}$$

where *A_p_* is the effective actuating area of the hydraulic cylinder, *y_c_* is the displacement of the hydraulic cylinder, *C_tp_* is the total leakage coefficient, defined by, *C_tp_* = *C_ip_* + *C_ep_*/2, and *C_ip_* and *C_ep_* are the internal and external leakage coefficients, respectively. *β_e_* is the hydraulic oil effective bulk modulus, and *V_t_* is the total volume of the hydraulic cylinder.

Considering that the mass of the hydraulic cylinder is insignificant in comparison to the mass of the barge and pier, it is omitted in the HABS model. By applying Newton’s force balance equation and neglecting both the internal friction of the hydraulic cylinder and the mass of the hydraulic oil, the output force exerted by the hydraulic cylinder on the payload is determined by the following expression:

(6) $$F_{c}=m_{c}(t)\frac{d^{2}y_{c}}{dt^{2}}+F_{d}(t)=-m_{b}\frac{d^{2}y_{b}}{dt^{2}}-C_{w}\frac{dy_{b}}{dt}-K_{w}y_{b}$$

where *F_c_* is the output force of the hydraulic cylinder, *y_b_* is the displacement of the barge and pier due to the load change and water fluid elasticity and buoyant effect, *m_b_* is the mass of the barge and pier, *C_w_* is the equivalent damper, *K_w_* is the equivalent spring stiffness, *m_c_*(*t*) is the equivalent mass, and the vehicle load is time-varying mass to the system, *y_c_* is the end displacement of the bridge in the point fixed with the hydraulic cylinder, *g* is the acceleration of gravity, and *F_d_*(*t*) is the equivalent disturbance force to the system due to the time-varying vehicle load effect on the bridge specimen and the vehicle-bridge coupled action.

**Figure 4:**
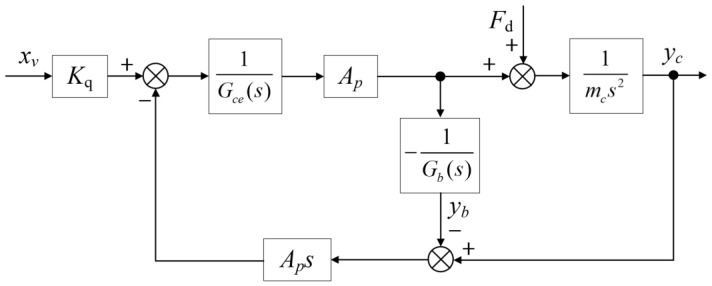
The open-loop control system block diagram of the HABS.

The block diagram of the position open-loop control system for the HABS, derived from Equations (1)–(6), is shown in Figure 4.

(7) $$\left\{\begin{array}{l} G_{ce}(s)=\frac{V_{t}}{4\beta_{e}}s+K_{c}+C_{tp}\\ G_{b}(s)=m_{b}s^{2}+C_{w}s+K_{w} \end{array}\right.$$

Using Equation (7) and the block diagram presented in Figure 4, the transfer function governing the actuator displacement is given by the following expression:

(8) $$Y_{c}=\frac{A_{p}K_{q}G_{b}(s)X_{v}+\left(G_{b}(s)G_{ce}(s)-{A_{p}}^{2}s\right)F_{d}}{\left(m_{c}s^{2}G_{ce}(s)+{A_{p}}^{2}s\right)G_{b}(s)+m_{c}{A_{p}}^{2}s^{3}}$$

Equation (8) demonstrates that the system’s output displacement *y_c_* is influenced by the mass of the barge and pier *m_b_*, the equivalent damper *C_w_*, and the equivalent spring stiffness *K_w_*. Additionally, the output displacement *y_c_* is affected by the equivalent disturbance force acting on the system *F_d_*(*t*). To address these factors, a servo control system is proposed in this study, aimed at minimising both displacement and acceleration, thereby ensuring the stability and safety of the flexible bridge specimen under vehicular loads. Based on Equation (8), the transfer functions describing the relationships between the servo valve displacement and the disturbance force in relation to the actuator displacement are formulated as follows:

(9) $$G_{ds}(s)=\frac{Y_{c}}{X_{v}}=\frac{A_{p}K_{q}G_{b}(s)}{\left(m_{c}s^{2}G_{ce}(s)+{A_{p}}^{2}s\right)G_{b}(s)+m_{c}{A_{p}}^{2}s^{3}}$$

(10) $$G_{fs}(s)=\frac{Y_{c}}{F_{d}}=\frac{G_{b}(s)G_{ce}(s)-{A_{p}}^{2}s}{\left(m_{c}s^{2}G_{ce}(s)+{A_{p}}^{2}s\right)G_{b}(s)+m_{c}{A_{p}}^{2}s^{3}}$$

## 3. Control Strategy Design

The controller design focuses on developing a robust system to mitigate the vibrations in the flexible bridge structure induced by external disturbances. By employing a multi-state observer and a PD controller, the design aims to optimise the system’s stability and performance. The proposed approach effectively shifts the closed-loop pole points to reduce resonant peaks, thus enhancing the control system’s ability to maintain the stability of the bridge under varying dynamic loads.

### 3.1. Position Closed-Loop Control Approach

Substituting Equation (7) into Equation (9) yields the following expression:

(11) $$G_{ds}(s)=\frac{Y_{c}}{X_{v}}=\frac{K_{q}/A_{p}\left(\frac{m_{b}}{K_{w}}s^{2}+\frac{C_{w}}{K_{w}}s+1\right)}{G_{m}(s)}$$

(12) $$\begin{array}{l} G_{m}(s)=\frac{m_{b}}{K_{w}}\frac{m_{c}V_{t}}{4\beta_{e}{A_{p}}^{2}}s^{5}+\left(\frac{m_{b}}{K_{w}}\frac{K_{ce}m_{c}}{{A_{p}}^{2}}+\frac{C_{w}}{K_{w}}\frac{m_{c}V_{t}}{4\beta_{e}{A_{p}}^{2}}\right)s^{4}\\ \;+\left(\frac{m_{c}V_{t}}{4\beta_{e}{A_{p}}^{2}}+\frac{C_{w}}{K_{w}}\frac{m_{c}K_{ce}}{{A_{p}}^{2}}+\frac{m_{b}+m_{c}}{K_{w}}\right)s^{3}+\left(\frac{m_{c}K_{ce}}{{A_{p}}^{2}}+\frac{C_{w}}{K_{w}}\right)s^{2}+s \end{array}$$

The following equations are defined as:

(13) $$\omega_{s}=\sqrt{\frac{4\beta_{e}{A_{p}}^{2}}{m_{c}V_{t}}};\;\xi_{s}=\frac{K_{ce}}{A_{p}}\sqrt{\frac{\beta_{e}m_{c}}{V_{t}}};\;\omega_{b}=\sqrt{\frac{K_{w}}{m_{b}}};\;\xi_{b}=\frac{C_{w}}{2\sqrt{m_{b}K_{w}}};\;\omega_{p}=\sqrt{\frac{K_{w}}{m_{b}+m_{c}}}$$

By substituting Equation (13) into Equation (12), the resulting expression is obtained as follows:

(14) $$G_{m}(s)=\frac{s^{5}}{{\omega_{b}}^{2}{\omega_{s}}^{2}}+\left(\frac{2\xi_{s}}{{\omega_{b}}^{2}\omega_{s}}+\frac{2\xi_{b}}{\omega_{b}{\omega_{s}}^{2}}\right)s^{4}+\left(\frac{4\xi_{s}\xi_{b}}{\omega_{b}\omega_{s}}+\frac{1}{{\omega_{s}}^{2}}+\frac{1}{{\omega_{p}}^{2}}\right)s^{3}+\left(\frac{2\xi_{s}}{\omega_{s}}+\frac{2\xi_{b}}{\omega_{b}}\right)s^{2}+s$$

Equation (14) represents a standard fifth-order system transfer function, which can be decomposed into two second-order transfer functions and an integrator element, as shown below:

(15) $$G_{m}(s)=s\left(\frac{s^{2}}{{\omega_{l}}^{2}}+\frac{2\xi_{l}}{\omega_{l}}s+1\right)\left(\frac{s^{2}}{{\omega_{h}}^{2}}+\frac{2\xi_{h}}{\omega_{h}}s+1\right)$$

where *ω_l_* is the lower natural frequency of the fifth-order system, *ω_h_* is the higher natural frequency of the fifth-order system, *ξ_l_* and *ξ_h_* are, respectively, the damping ratio of the system *G_m_*(*s*).

Given the existence of the following equation *ω_s_* = *ω_h_*, Equations (14) and (15) can then be described as follows:

(16) $$G_{m}(s)=s\left(\frac{s^{2}}{{\omega_{s}}^{2}}+\frac{2\xi_{s}}{\omega_{s}}s+1\right)\left(\frac{s^{2}}{{\omega_{b}}^{2}}+\frac{2\xi_{b}}{\omega_{b}}s+1\right)$$

The corresponding Equation (11) can be simplified as follows:

(17) $$G_{ds}(s)=\frac{Y_{c}}{X_{v}}=\frac{K_{q}/A_{p}}{s\left(\frac{s^{2}}{{\omega_{s}}^{2}}+\frac{2\xi_{s}}{\omega_{s}}s+1\right)}$$

**Figure 5:**
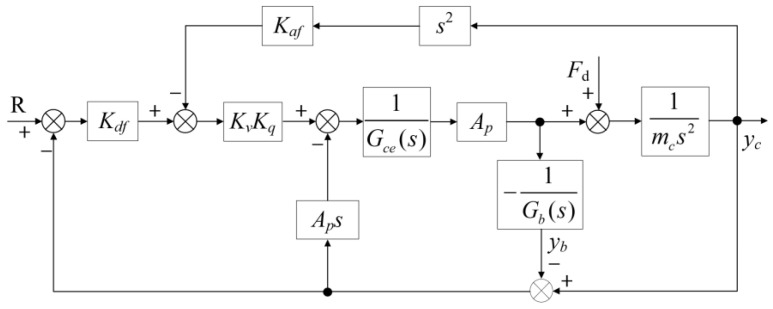
The position close-loop control scheme based on traditional TVC.

Equation (17) describes a standard hydraulic closed-loop control system without considering the effects of a flexible foundation. In the system studied here, however, the equivalent mass-spring-damping effect introduced by the water generates a coupled natural frequency, which transforms the system into a fifth-order system, as shown in Equation (16). To enhance control performance in such complex systems, Han [45] introduced the three-variable controller (TVC), which has proven to be an effective solution for electro-hydraulic position closed-loop servo control systems. The TVC’s effectiveness and feasibility have been validated extensively through both theoretical research and practical engineering applications. Figure 5 illustrates the integration of the TVC within the HABS, showcasing its role in improving the system’s dynamic response and stability under varying operating conditions.

As shown in Figure 5, the measured displacement of the hydraulic cylinder corresponds to the relative displacement between the bridge end and the hydraulic cylinder, rather than the absolute displacement of the bridge itself. In contrast, the acceleration measured by the sensor represents the absolute value. The transfer function governing the position closed-loop control system, as illustrated in Figure 5, is given by the following expression:

(18) $$G_{c}(s)=\frac{Y_{c}}{R}=\frac{\left(\frac{s^{2}}{{\omega_{b}}^{2}}+\frac{2\xi_{b}}{\omega_{b}}s+1\right)}{\left(\frac{s}{\omega_{mc}}+1\right)\left(\frac{s^{2}}{{\omega_{lc}}^{2}}+\frac{2\xi_{lc}}{\omega_{lc}}s+1\right)\left(\frac{s^{2}}{{\omega_{hc}}^{2}}+\frac{2\xi_{hc}}{\omega_{hc}}s+1\right)}$$

Without considering the flexible foundation, the transfer function of the position closed-loop control system is described as follows:

(19) $$G_{cg}(s)=\frac{Y_{c}}{R}=\frac{1}{\left(\frac{s}{\omega_{cg}}+1\right)\left(\frac{s^{2}}{{\omega_{nc}}^{2}}+\frac{2\xi_{nc}}{\omega_{nc}}s+1\right)}$$

**Figure 6:**
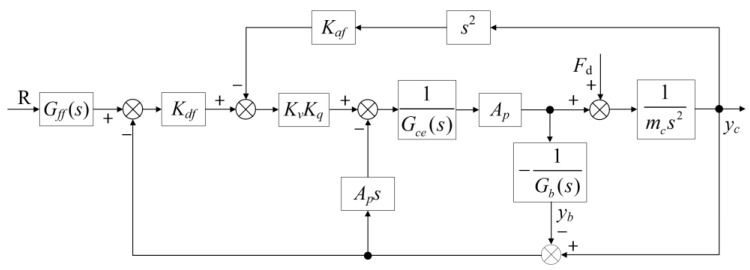
The position close-loop control based on improved TVC.

A comparison between Equations (18) and (19) indicates that the introduction of a flexible foundation adds both a second-order differential system and a second-order oscillation system, in contrast to the simpler dynamics of a rigid foundation system. The flexible foundation reduces the natural frequency and damping ratio of the control system, which can compromise stability and control performance. These changes necessitate the implementation of a more sophisticated control strategy to address the impact of the foundation’s flexibility. In such systems, feedback control can sometimes lead to instability, particularly in the presence of external disturbances and varying flexible loads. To address these challenges, an improved control strategy is proposed, incorporating an estimated TVC feedforward approach. This approach aims to mitigate the adverse effects introduced by the flexible foundation while ensuring enhanced stability and performance. The corresponding control block diagram is presented in Figure 6.

The objective of the estimated TVC feedforward controller is to eliminate the zero and pole points near the imaginary axis of the system described by Equation (18), which are introduced by the flexible foundation. These points contribute to resonant and anti-resonant peaks, leading to a decline in the system’s overall control performance. Considering the preceding analysis, the following feedforward controller is proposed:

(20) $$G_{ff}(s)=\frac{\frac{s^{2}}{{\omega_{lce}}^{2}}+\frac{2\xi_{lce}}{\omega_{lce}}s+1}{\frac{s^{2}}{{\omega_{be}}^{2}}+\frac{2\xi_{be}}{\omega_{be}}s+1}$$

Using Equations (18) and (20), and Figure 6 as references, the transfer function of the system that incorporates the estimated TVC feedforward controller is expressed as follows:

(21) $$G_{cf}(s)=\frac{\left(\frac{s^{2}}{{\omega_{b}}^{2}}+\frac{2\xi_{b}}{\omega_{b}}s+1\right)\left(\frac{s^{2}}{{\omega_{lce}}^{2}}+\frac{2\xi_{lce}}{\omega_{lce}}s+1\right)}{\left(\frac{s}{\omega_{mc}}+1\right)\left(\frac{s^{2}}{{\omega_{lc}}^{2}}+\frac{2\xi_{lc}}{\omega_{lc}}s+1\right)\left(\frac{s^{2}}{{\omega_{hc}}^{2}}+\frac{2\xi_{hc}}{\omega_{hc}}s+1\right)\left(\frac{s^{2}}{{\omega_{be}}^{2}}+\frac{2\xi_{be}}{\omega_{be}}s+1\right)}$$

Equation (21) clearly demonstrates that the feedforward controller can eliminate a pair of zero and pole points near the imaginary axis of the closed-loop control system, provided the control parameters *G_ff_*(*s*) are properly tuned. By optimising these parameters: *ω_be_* = *ω_b_*, *ξ_be_* = *ξ_b_*, *ω_lce_* = *ω_lc_*, *ξ_lce_* = *ξ_lc_*, the system attains optimal performance, and Equation (21) simplifies to the following expression:

(22) $$G_{cfs}(s)=\frac{1}{\left(\frac{s}{\omega_{mc}}+1\right)\left(\frac{s^{2}}{{\omega_{hc}}^{2}}+\frac{2\xi_{hc}}{\omega_{hc}}s+1\right)}$$

Equation (22) indicates that the resonant and anti-resonant peaks at lower natural frequencies, induced by the flexible foundation, are effectively cancelled. Consequently, the control system’s performance is significantly enhanced. It should be noted that due to variations in parameters and the inherent complexity of the system, achieving an accurate model can be challenging. However, when the parameters of the estimated feedforward controller are sufficiently close to the optimal solution, the resonant and anti-resonant peaks at lower frequencies are greatly diminished, leading to a marked improvement in system performance. Importantly, the parameter adjustments do not compromise system stability, which is essential for maintaining the reliability and robustness of the control system.

The settings for the TVC were determined through an iterative process combining theoretical analysis and experimental testing. Initial parameters were selected based on the system’s dynamic characteristics, such as natural frequencies and damping ratios. These parameters were further refined using a root locus method to ensure stability and optimal performance. To validate the settings, the controller was tested under a range of scenarios, including varying vehicle loads and fluctuating water levels, using both sine wave and random disturbance inputs.

### 3.2. Disturbance Observers and Vibration Control Strategies

**Figure 7:**
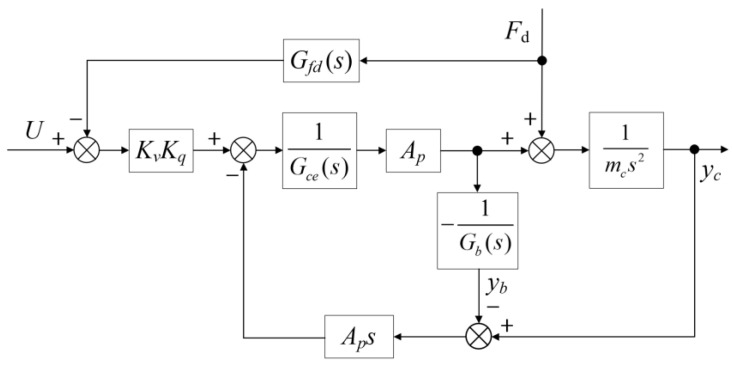
The feedforward compensation disturbance control scheme.

In the proposed HABS, the force generated by a vehicle crossing the bridge is modelled as a significant, time-varying disturbance. This disturbance induces considerable vibrations in the flexible bridge structure, which in turn can limit the permissible vehicle speed and pose potential safety risks. To address this challenge, a compensation controller based on a disturbance observer is employed to reduce the impact of the disturbance on the bridge. In addition, a multi-state observer featuring a PD controller is implemented to further mitigate the vibrations and enhance system stability. The combination of these controllers aims to improve the overall performance of the bridge, ensuring smoother traffic flow and maintaining safety standards. The block diagram illustrating the proposed feedforward compensation and disturbance control scheme is presented in Figure 7.

Using Equation (7) and Figure 7 as references, the transfer function from the input voltage *U* to the displacement output *y_c_* is given by the following expression:

(23) $$Y_{c}(s)=\frac{K_{q}K_{v}A_{p}G_{b}(s)U+\left(G_{ce}(s)G_{b}(s)-K_{q}K_{v}A_{p}G_{fd}(s)G_{b}(s)+{A_{p}}^{2}s\right)F_{d}}{G_{ce}(s)G_{b}(s)m_{c}s^{2}+{A_{p}}^{2}s\left(m_{c}s^{2}+G_{b}(s)\right)}$$

Equation (23) indicates that the feedforward compensation controller must satisfy the following condition to ensure that the system’s output displacement *y_c_* remains unaffected by external forces:

(24) $$G_{fd}(s)=\frac{s}{K_{q}K_{v}\left(m_{b}s^{2}+C_{w}s+K_{w}\right)}+\frac{V_{t}}{K_{q}K_{v}A_{p}4\beta_{e}}s+\frac{K_{ce}}{K_{q}K_{v}A_{p}}$$

Equation (24) reveals that the last two terms of the feedforward compensation controller act as a proportional-plus-integral (PI) controller, while the first term functions as an integral controller linked to the flexible foundation. Given these conditions, Equation (23) can be simplified as follows:

(25) $$Y_{c}(s)=\frac{K_{q}K_{v}A_{p}G_{b}(s)U}{G_{ce}(s)G_{b}(s)m_{c}s^{2}+{A_{p}}^{2}s\left(m_{c}s^{2}+G_{b}(s)\right)}$$

**Figure 8:**
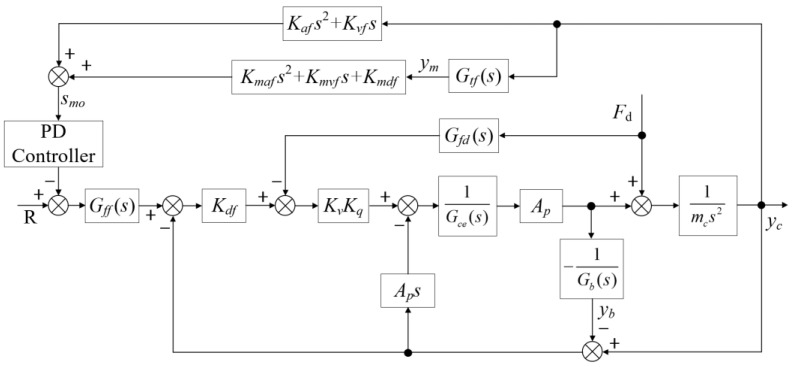
The total combined control scheme of the HABS.

In Figure 8, the following definitions are provided:

(26) $$\left\{\begin{array}{l} G_{1s}(s)=K_{af}s^{2}+K_{vf}s\\ G_{2s}(s)=K_{maf}s^{2}+K_{mvf}s+K_{dvf} \end{array}\right.$$

Figure 8 presents the block diagram of the proposed multi-state observer, which utilises a PD controller to mitigate bridge vibrations. The transfer function *G_ff_*(*s*) typically represents the relationship between the response at the midpoint of the flexible bridge and the response at its end, which is characterised as at least a second-order system. The PD controller is defined as *G_fz_*(*s*). Drawing from Figure 8 and Equations (22), (25) and (26), the transfer function from the input *R* to the output *y_c_* in Figure 8 can be derived as follows:

(27) $$G_{cfw}(s)=\frac{1}{\left(\frac{s}{\omega_{mc}}+1\right)\left(\frac{s^{2}}{{\omega_{hc}}^{2}}+\frac{2\xi_{hc}}{\omega_{hc}}s+1\right)+\left(G_{1s}(s)+G_{tf}(s)G_{2s}(s)\right)G_{fz}(s)}$$

By combining Equation (26) with the PD controller, the transfer function of the control system, represented by Equation (27), can be described as follows:

(28) $$G_{cfw}(s)=\frac{1}{\left(\frac{s}{\omega_{mc}}+1\right)\left(\frac{s^{2}}{{\omega_{hc}}^{2}}+\frac{2\xi_{hc}}{\omega_{hc}}s+1\right)+\left(\left(K_{maf}s^{2}+K_{mvf}s+K_{dvf}\right)G_{tf}(s)+K_{af}s^{2}+K_{vf}s\right)\left({\tilde{K}}_{D}s+{\tilde{K}}_{p}\right)}$$

where ${\tilde{K}}_{p}\;\mathrm{and}\;{\tilde{K}}_{D}$ represent the control parameters of the PD controller. From Equation (28), it is evident that the control system can shift the closed-loop pole points, which effectively reduces the peak amplitude at the resonant natural frequency of the flexible bridge. This reduction addresses the primary factor influencing the bridge’s vibration response.

The proposed control strategy, which integrates a PD controller and multi-state observer, provides distinct advantages compared to traditional approaches. Conventional PID controllers, while effective in many applications, often exhibit limitations in handling rapidly changing dynamic conditions, such as fluctuating vehicle loads and water levels. Advanced methods like ADRC can address these issues but typically involve higher computational complexity, which can pose challenges for real-time applications. In contrast, the PD controller delivers an efficient and reliable performance with reduced computational demands, while the multi-state observer enhances the system’s ability to suppress disturbances and minimise vibrations. This combination achieves a robust balance between operational performance and practical feasibility, making it particularly well-suited for dynamic environments like floating bridges.

## 4. Co-Simulation Study

Section 4 provides an in-depth co-simulation study to evaluate the proposed Hydraulic Adaptive Bearing System (HABS). This study integrates MSC Adams for mechanical modelling with MATLAB/Simulink for hydraulic and control system simulations, forming a comprehensive framework for performance analysis. A scaled model of the flexible beam is employed to replicate real-world dynamics, with hydraulic cylinders simulating external disturbances and water wave effects, while a spring represents the elastic force of the water. The simulation tests explore various scenarios to assess the system’s effectiveness in managing bearing displacement and controlling vibrations. The results confirm that the proposed HABS significantly improves control performance and demonstrates its applicability in real-world situations.

### 4.1. Co-Simulation Model Development

**Figure 9:**
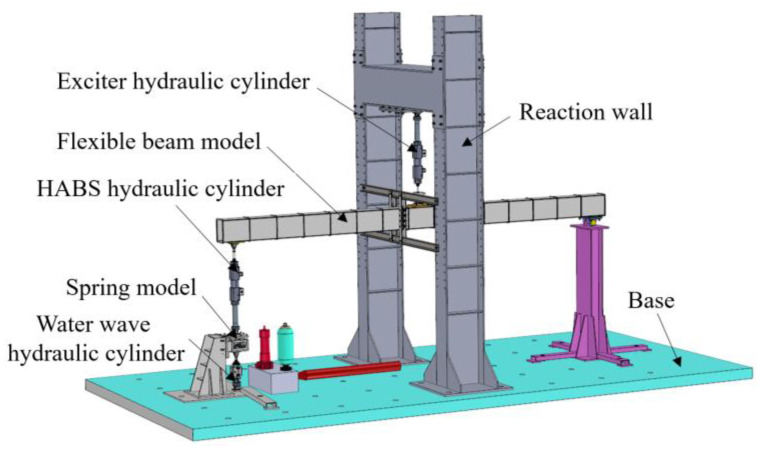
The mechanical model of the HABS.

To validate the performance of the proposed HABS, a comprehensive co-simulation model and a test-rig setup were developed. Figure 9 presents the complete mechanical model of the HABS, which is based on a 1:15 scale flexible beam that accurately simulates the behaviour of the full-scale 87-type flexible beam. Equivalent modal analysis was employed to replicate the beam’s dynamic characteristics, ensuring that the scaled model mirrors the structural responses of the full-scale system under various operational conditions. The self-adaptive bearing control system is modelled through a hydraulic cylinder governed by a servo valve. To simulate external disturbance forces, an exciter hydraulic cylinder is introduced, which is also controlled by a servo valve and operates using a force closed-loop control strategy. It allows for the realistic simulation of varying external loads, such as those caused by vehicle traffic. Additionally, water level fluctuations are simulated using a wave hydraulic cylinder that operates under a position closed-loop control system, again controlled by a servo valve. To account for the elastic properties of the water, a spring element is incorporated into the model to mimic the hydro-elastic forces exerted on the bridge. The combination of these elements provides a realistic and dynamic simulation environment, allowing for in-depth analysis of the HABS performance under various conditions. Key system parameters used in the co-simulation are provided in Table 1.

### 4.2. Performance Analysis and Results

**Figure 10:**
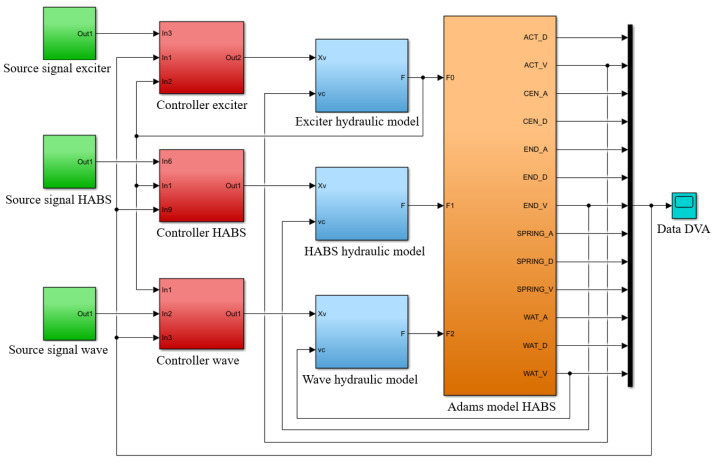
The co-simulation model of the HABS.

The HABS simulation model was developed using a co-simulation approach, integrating MSC Adams and MATLAB/Simulink to capture the full dynamics of the system. The flexible beam, which forms a key component of the HABS, was initially designed and analysed in ANSYS to model its mechanical properties and dynamic behaviour. After conducting the necessary modal and structural analyses, the flexible beam model was imported into MSC Adams, where the mechanical components of the system were fully modelled, enabling simulation of the system’s physical interactions and dynamic responses to various forces. The hydraulic and control system models, including the servo valve mechanisms and control loops, were developed in MATLAB/Simulink. These models incorporate the control algorithms and hydraulic dynamics necessary for simulating the behaviour of the HABS under real-world conditions. Once both the mechanical and hydraulic models were established, they were integrated into a co-simulation environment using MSC Adams and MATLAB/Simulink. This setup facilitates the seamless interaction between the mechanical components and the hydraulic control system, ensuring that the dynamic responses are accurately represented in a unified prediction work. The co-simulation process and system integration are shown in Figure 10.

(A) Displacement Compensation Tests

**Figure 11:**
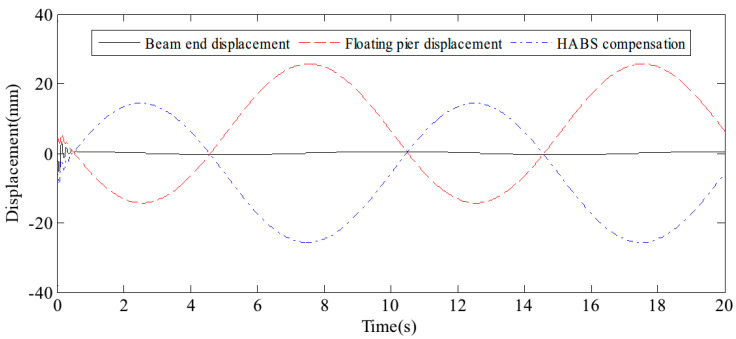
The bearing displacement control effect under sine signal tests.

**Figure 12:**
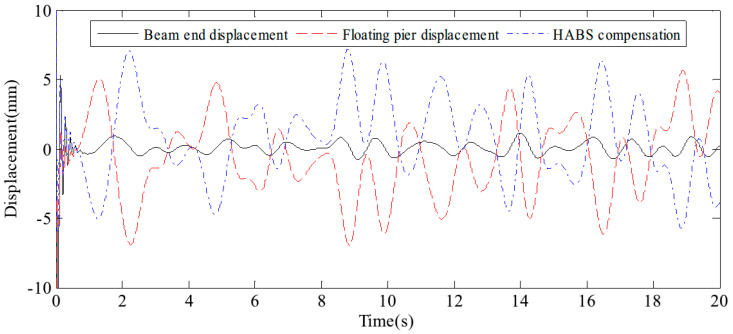
The bearing displacement control effect under random signal tests.

The external variations in vehicle load on the flexible beam, coupled with the influence of water waves, both contribute significantly to system displacement. To assess the bearing displacement and evaluate the effectiveness of the control system, sine and random signals were applied to the water hydraulic cylinder control system. The detected signals simulate the dynamic forces exerted by environmental conditions and vehicle movement. It results in changes in bearing displacement, as well as the effectiveness of the control measures, as shown in Figure 11 and Figure 12. The test results confirm that the proposed HABS successfully compensates for bearing displacement, maintaining stability and mitigating the adverse effects caused by both vehicle load variations and wave action.

(B) Vibration Control Results

**Figure 13:**
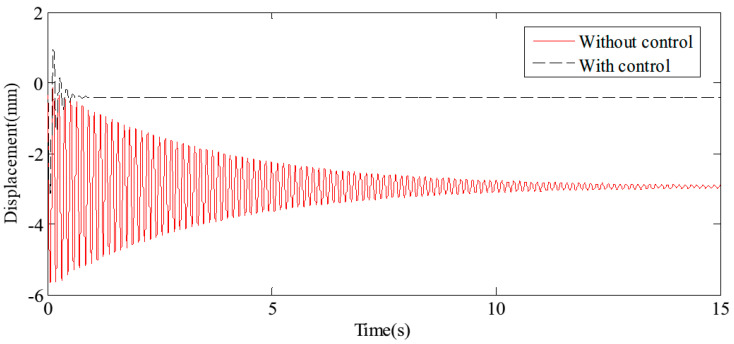
The displacement curves in the beam middle under free vibration.

**Figure 14:**
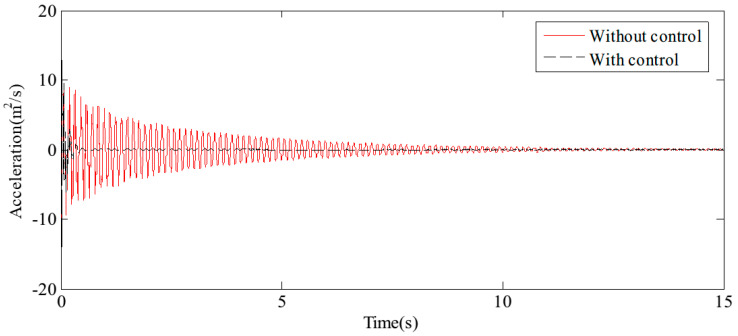
The acceleration curves in the beam middle under free vibration.

The midpoint of the flexible beam experiences the most pronounced vibration when vehicles pass over it, making this location the critical reference point for the analysis. The results of free vibration control, specifically the displacement and acceleration at the midpoint, are illustrated in Figure 13 and Figure 14, respectively. The findings indicate that, with the application of the proposed HABS compensation control method, the vibration attenuation time is significantly reduced from 15 s to approximately 0.6 s. Furthermore, the vibration level decreases from 100% to just 5%, demonstrating a substantial improvement in control effectiveness. It is important to note that the displacement at the midpoint stabilises at a constant value rather than at zero, due to the combined effects of gravity and the natural bending of the flexible beam.

**Figure 15:**
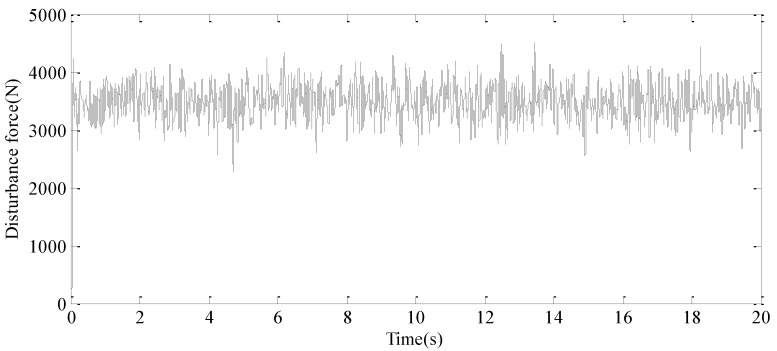
The random external vehicle force signal.

**Figure 16:**
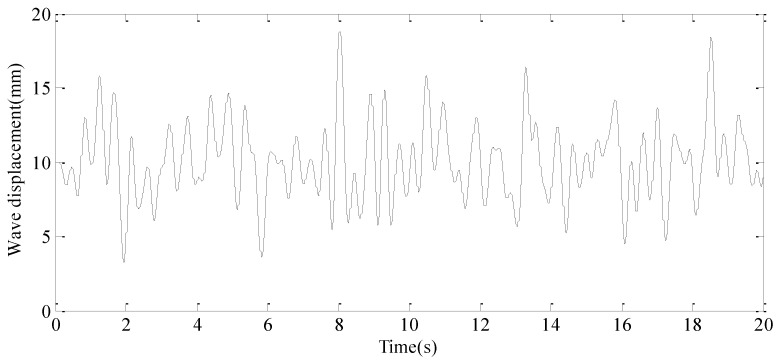
The random disturbance displacement signal.

A random external vehicle force signal, with a frequency range of 0.1 Hz to 30 Hz, is applied to the exciter hydraulic cylinder control system to simulate external disturbance forces. Concurrently, a random displacement disturbance signal, ranging from 0.1 Hz to 3 Hz, is introduced to the wave hydraulic cylinder control system to replicate the effects of random water wave motion. The resulting system responses are depicted in Figure 15 and Figure 16, respectively.

**Figure 17:**
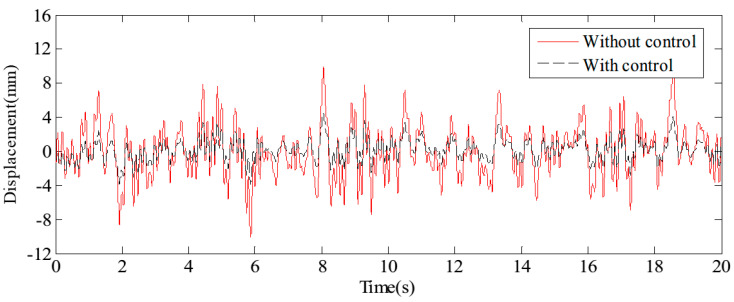
The displacement curves in the beam end.

**Figure 18:**
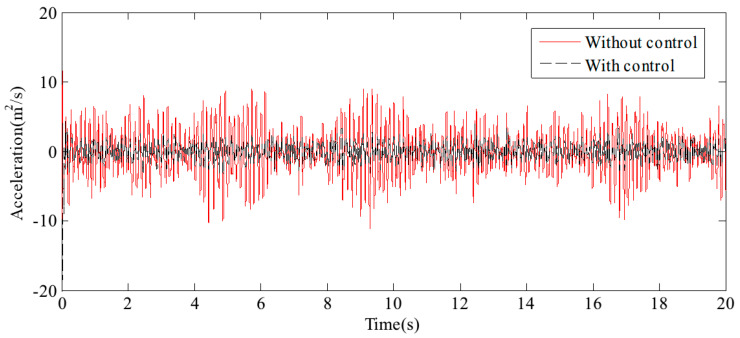
The acceleration curves in the beam end.

**Figure 19:**
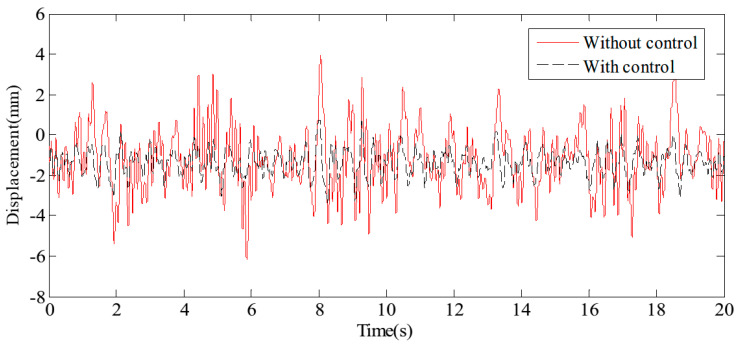
The displacement curves in the beam middle.

**Figure 20:**
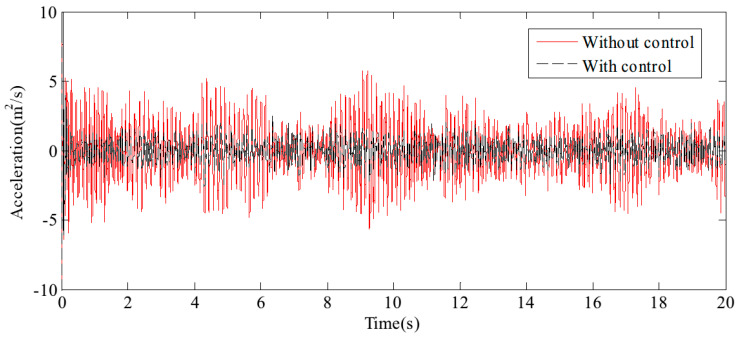
The acceleration curves in the beam middle.

**Figure 21:**
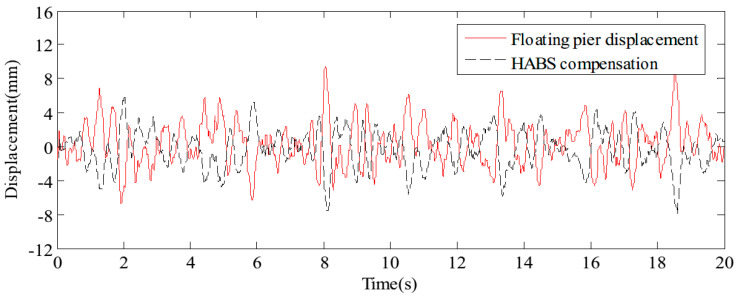
The HABS compensation control signal.

Figure 17 and Figure 18 display the displacement and acceleration response signals at the end point of the flexible beam, comparing the results with and without the proposed HABS control method. Similarly, Figure 19 and Figure 20 illustrate the displacement and acceleration response signals at the midpoint of the flexible beam, under both control scenarios. The corresponding statistical data, summarising the system’s performance with and without the HABS control method, are presented in Table 2. Additionally, Figure 21 shows the compensation control signal generated by the HABS, along with the displacement signal of the floating pier during the testing process.

The results indicate that, without the compensation control scheme, the displacement at the end of the beam ranges from −10.14 mm to 10.27 mm. With the proposed control method, however, the displacement range is significantly reduced from −4.68 mm to 5.62 mm. The root mean square (RMS) value of the displacement at the beam’s end is decreased by 52.88%, while the maximum displacement is reduced by 54.72%. Furthermore, the RMS value of the acceleration at the beam’s midpoint is lowered by 43.55%, and the maximum acceleration is reduced by 46.10% when the control method is applied. These simulation results demonstrate the feasibility and effectiveness of the proposed HABS and its control strategy.

The simulation study highlights several key improvements, including the integration of the Hydraulic Self-Adaptive Bearing System (HABS) with a compensation control scheme that effectively mitigates external disturbances such as vehicle loads and water wave motion. The control method demonstrated significant reductions in displacement and acceleration, along with improved vibration attenuation. The co-simulation approach, combining MSC Adams and MATLAB/Simulink, offered a detailed evaluation of the system’s performance under dynamic conditions. The proposed HABS control scheme also proved robust in managing random external forces and disturbances, underscoring its practical applicability to real-world scenarios.

## 5. Test-Rig Setup and Validation

A test-rig setup was created to test the proposed HABS and its control scheme using a scaled model. The system, featuring hydraulic cylinders and sensors, effectively simulates real-world conditions, including disturbances and water level variations. Utilising MATLAB/Simulink for control and data acquisition, the measured results validate the effectiveness of the proposed control method. The findings show significant improvements in beam stability and vibration reduction, revealing the novel contributions of the control strategy in enhancing overall system performance and stability.

### 5.1. Prototype Development

#### 5.1.1. Scaling and Dynamic Similarity Principles

**Figure 22:**
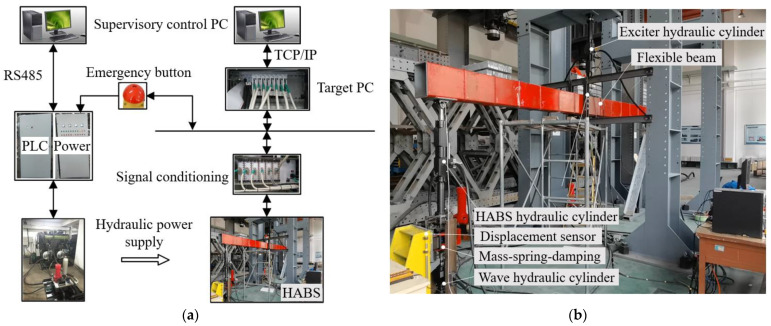
Prototype of the proposed HABS; (**a**) Prototype of the proposed HABS, (**b**) Key components of the HABS.

To further validate the feasibility and effectiveness of the proposed HABS and its control scheme, a test-rig was developed using a 1:15 scale model, as shown in Figure 22. The 1:15 scale model was designed using principles of dynamic similarity to ensure that the behaviour of the prototype accurately reflects that of a full-size bridge under comparable conditions. Parameters such as material properties, load distribution, and hydraulic response were carefully scaled based on established similitude laws. The proposed approach ensures that critical dynamics, including displacement, vibration, and adaptive control behaviours, remain consistent across scales. While certain practical factors, such as environmental complexities, vary in real-world applications, the scaled model provides a reliable foundation for validating the performance and feasibility of the proposed HABS in full-size implementations. Given the limitations of the scale model, a disturbance force generator, operating on a force closed-loop control system, was utilised in place of a full vehicle model to simulate external forces. Additionally, the wave hydraulic cylinder, governed by a servo valve with position closed-loop control, was employed to simulate water level variations, while a spring was incorporated to replicate the elastic forces exerted by the water.

#### 5.1.2. System Components and Setup

The prototype is constructed to comprehensively validate the performance of the proposed Hydraulic Self-Adaptive Bearing System (HABS) and its control scheme. The system includes a range of components: a flexible beam specimen, hydraulic cylinders, Moog servo valves, LVDT displacement sensors, acceleration sensors, a force sensor, a reaction base, spherical hinges, and a hydraulic power supply. The Moog servo valves, featuring a flow capacity of 38 L/min at a supply pressure of 7 MPa, play a crucial role in controlling the hydraulic cylinders. These cylinders, designed with a bore of 60 mm and a rod diameter of 50 mm, generate the driving force necessary for simulating real-world dynamic loads. Displacement is measured by LVDT sensors attached to the hydraulic cylinders, while acceleration sensors from Endevco are mounted at both the midpoint and endpoint of the flexible beam to capture detailed acceleration signals, which are essential for evaluating system vibration response.

A key development in this test-rig is the integration of a force closed-loop control mechanism. This is implemented through a hydraulic cylinder located at the midpoint of the flexible beam, which is controlled by a servo valve to simulate varying external disturbance forces, such as those induced by vehicle loads. Another innovative aspect is the hydraulic self-adaptive bearing system itself, positioned at the beam’s endpoint. This system utilises a hydraulic cylinder, connected in a series with a spring, and operates under position closed-loop control. The servo valve in this configuration dynamically adjusts the system’s response, providing real-time vibration compensation—a critical feature for stabilising the flexible bridge structure. Additionally, a wave hydraulic cylinder, also located by a servo valve under position closed-loop control, is employed to simulate water wave motion. This setup allows for a realistic replication of fluctuating environmental forces, further enhancing the robustness of the system validation. The novelty of this test-rig lies in its ability to accurately simulate complex, multi-axis disturbances and to assess the performance of the HABS in mitigating these effects, ensuring the stability and safety of flexible bridge structures in dynamic environments. The key parameters of the test-rig setup, including detailed system specifications, are summarised in Table 1.

The hardware architecture of the control system, as shown in Figure 22, is implemented using the xPC Target platform, which facilitates rapid prototyping within MATLAB/Simulink. The control system and data acquisition programmes are initially designed in MATLAB/Simulink and compiled with Microsoft Visual Studio.NET on the host PC. These compiled programmes are then transferred to the target PC for execution, enabling real-time control. The control hardware setup includes an Advantech IPC-510 industrial controller, equipped with PCI-1716 and PCI-9114 cards, along with a host PC and various peripheral components for signal processing.

The system’s feedback signals, including displacement, acceleration, and force, are first processed through a conditioning module that converts and amplifies them into a standard voltage range of −10 V to 10 V. These signals are then captured by the 16-bit Advantech A/D card (PCI-9114) installed on the target PC, allowing for high-precision data acquisition. The control system’s analogue outputs are generated by the D/A acquisition board (PCI-1716), which converts digital control signals back into analogue form. These analogue signals are subsequently routed through a signal conditioning module to the servo valve, enabling precise actuation of the hydraulic cylinders within the system. This architecture ensures accurate and responsive control, crucial for the real-time management of the hydraulic self-adaptive bearing system and its ability to mitigate disturbances effectively.

### 5.2. The Experiment Tests Research

#### 5.2.1. Sine Signal Test

**Figure 23:**
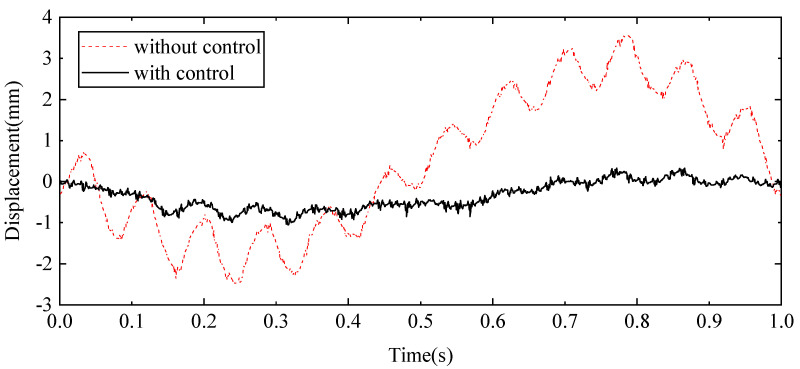
The displacement curves in the beam end under sine signal tests.

**Figure 24:**
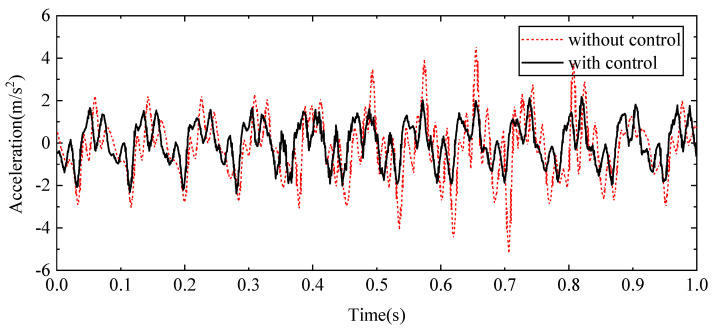
The acceleration curves in the beam middle under sine signal tests.

To evaluate the performance of the proposed control scheme, a 12 Hz sine signal was applied to the exciter hydraulic cylinder control system to simulate high-frequency external disturbances. Concurrently, a 1 Hz sine signal was applied to the wave hydraulic cylinder control system to replicate low-frequency water wave motions. The resulting displacement response at the beam’s endpoint and the acceleration response at the midpoint of the beam were recorded. These response curves, presented in Figure 23 and Figure 24, respectively, show the effects of the proposed control method compared to the baseline system without the control scheme.

Figure 23 shows how the endpoint displacement is influenced by the applied sine signals, highlighting the differences in response when the control scheme is active versus when it is not. Similarly, Figure 24 shows the acceleration at the beam’s midpoint, providing insights into the system’s dynamic behaviour under the same conditions. The effectiveness of the control method is further quantified in Table 3, which summarises key statistical data including displacement and acceleration metrics. These data demonstrate the control scheme’s ability to reduce vibrations and stabilise the system, confirming its effectiveness in managing both high-frequency disturbances and low-frequency wave motions.

The measured results demonstrate a substantial improvement in system performance when employing the proposed compensation control method. Without the compensation control, the displacement at the beam’s endpoint fluctuates between −2.50 mm and 3.56 mm. In contrast, with the application of the proposed control method, the range is reduced to between −0.05 mm and 0.33 mm. The reduction in displacement indicates a significant enhancement in the control system’s ability to stabilise the beam. Specifically, the root mean square (RMS) value of the endpoint displacement is decreased by 80.27%, reflecting a more consistent and minimised deviation from the equilibrium position. Furthermore, the maximum displacement is reduced by 62.73%, showing a marked decrease in extreme fluctuations. In addition to the improvements in displacement, the proposed control method also positively impacts the acceleration at the beam’s midpoint. The RMS value of acceleration is decreased by 33.01%, which suggests a smoother performance with less variability in the acceleration profile. The maximum acceleration is reduced by 54.19%, indicating that the control method effectively minimises high-intensity dynamic responses. The results reveal the effectiveness of the proposed HABS control method in significantly enhancing the stability and performance of the flexible beam, demonstrating its capability to mitigate vibrations and improve overall system reliability.

#### 5.2.2. Random Signal Test

**Figure 25:**
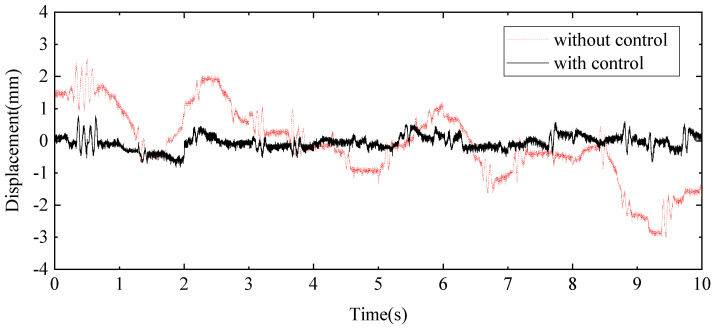
The displacement curves in the beam end under random signal tests.

**Figure 26:**
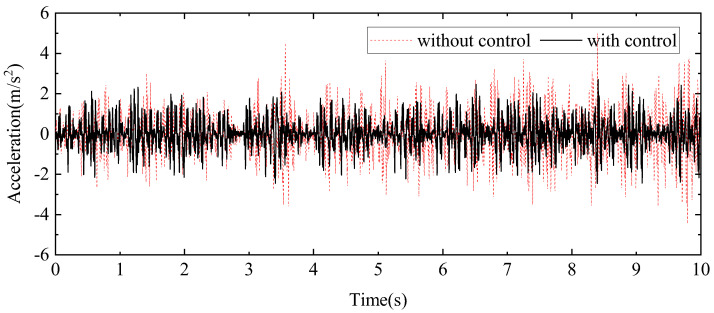
The acceleration curves in the beam middle under random signal tests.

A random signal with a frequency range of 0.1 Hz to 20 Hz is applied to the exciter hydraulic cylinder control system to simulate external disturbance forces, while a separate random signal with a frequency range of 0.1 Hz to 1 Hz is applied to the wave hydraulic cylinder control system to water wave motion. The measured results are illustrated through displacement response curves at the endpoint of the beam, shown in Figure 25, and acceleration response curves at the midpoint of the beam, presented in Figure 26. These curves provide a detailed view of the system’s behaviour under varying signal conditions. For a comprehensive analysis of the control effects, the typical statistical data are summarised in Table 4, highlighting the performance improvements achieved by the proposed HABS and control strategies. The data include key metrics such as displacement and acceleration reductions, which emphasise the effectiveness of the control system in managing dynamic disturbances and improving the stability and performance of the flexible beam.

The measured results demonstrate a marked improvement in system performance with the implementation of the proposed control method. Without compensation control, the displacement at the endpoint of the beam fluctuates between −3.02 mm and 2.58 mm. In contrast, when the proposed control method is applied, this range is reduced to between −0.82 mm and 0.78 mm. This substantial reduction in displacement, by 79.23% in terms of the root mean square (RMS) value and by 72.83% in the maximum displacement, indicates a significant enhancement in the system’s ability to manage endpoint displacements effectively. Similarly, the application of the proposed control method yields considerable improvements in acceleration measurements. The RMS value of the acceleration at the midpoint of the beam decreased by 30.32%, reflecting a reduction in the overall vibration intensity. Furthermore, the maximum acceleration at this point is reduced by 50.67%, highlighting the control method’s effectiveness in mitigating peak accelerative forces. These results indicate the proposed control method’s ability to substantially reduce both displacement and acceleration, enhancing the overall stability and performance of the flexible beam system.

The comparison between the simulation and experimental results reveals a strong correlation in the observed dynamic behaviour, particularly in displacement and acceleration trends at critical points of the flexible beam. Similar responses were identified under various input conditions, including sine wave and random disturbances. The strong agreement shows that the simulation model replicates the experimental setup, effectively capturing key performance characteristics and supporting its validity for further analysis and practical applications.

The measured results show significant reductions in displacement and vibration, which are critical for improving the performance of floating bridges under real-world conditions. For instance, the proposed HABS can be applied in emergency bridge repairs following natural disasters like floods, where fluctuating water levels and variable vehicle loads pose severe challenges. The ability to maintain minimal bridge displacement and adapt to dynamic conditions ensures safer and more stable traffic operations. In various dynamic applications, where floating bridges are often deployed in complex terrains and under time-sensitive conditions, the HABS offers reliability and quick adaptability, enabling efficient transportation of heavy vehicles and equipment. These capabilities make the HABS an ideal choice for improving the stability, durability, and operational efficiency of modern floating bridge systems.

## 6. Conclusions

This paper introduces a novel Hydraulic Self-Adaptive Bearing System (HABS) for elevated floating bridges, highlighting several significant advancements. A key innovation is the development of a real-time control strategy featuring an enhanced three-variable controller (TVC), which addresses the flexibility of the basic support system due to hydro-elastic effects. This improvement allows for more precise control in response to dynamic bridge conditions. Additionally, the paper proposes a multi-state observer control strategy that integrates a PD controller with feedforward compensation, effectively reducing bridge vibrations and improving overall system stability. The use of a co-simulation model, combining MATLAB/Simulink with MSC Adams, provides a robust framework for simulating various operating conditions and refining system performance. The test-rig, developed using xPC Target rapid prototyping techniques, offers a high level of precision in testing and real-time performance assessment. Through extensive comparative experiments with sine and random signals, the results demonstrate substantial improvements: the HABS reduces system displacement by 72% and root mean square (RMS) values by approximately 79%, while acceleration RMS and maximum values decrease by 33% and 54%, respectively. These findings highlight the effectiveness of the proposed HABS and control strategies in significantly enhancing bridge stability and reducing vibrations, marking a substantial advancement in hydraulic bearing systems for elevated bridges.

While the HABS shows significant improvements in stability and vibration mitigation, certain challenges remain. For instance, the reliance on precise hydraulic control systems may increase the complexity of implementation and maintenance, especially in harsh or remote environments. Additionally, the scalability of the system for larger infrastructure applications may require further validation under real-world conditions. Future work will focus on addressing these challenges by exploring robust control strategies and cost-effective design enhancements to ensure broader applicability and reliability.

## Figures and Tables

**Table 1:** The main system parameters of the HABS.

Parameters	Value	Parameters	Value
Effective area of hydraulic cylinder	8.64 cm^2^	Mass of 87-type beam model	400 Kg
Stroke of hydraulic cylinder	±100 mm	Pressure of hydraulic source	21 MPa
Rated flow of servo valve	64 L/min	Stiffness of spring model	40 kg/mm
Natural frequency of servo valve	200 Hz	Load range of spring stiffness	800–1200 Kg
Damping ratio of servo valve	0.6	First mode frequency of beam	31 Hz
Density of hydraulic oil	845 kg/m^3^	Size of beam model	0.2 × 0.3 × 4 m
Elasticity bulk modulus	6.9 × 10^8^ Pa	Step time of control system	1 ms

**Table 2:** The statistics data of the test results.

mm/(m∙s^−2^)	RMS			The Maximum Value(abs)		
	Without Control	With Control	Control Effect	Without Control	With Control	Control Effect
End displacement	2.95	1.56	52.88%	10.27	5.62	54.72%
End acceleration	3.17	1.07	33.75%	11.17	3.93	35.18%
Middle displacement	1.93	1.36	64.98%	6.16	3.39	55.03%
Middle acceleration	1.86	0.81	43.55%	5.77	2.66	46.10%

**Table 3:** The statistics data under sine signal tests.

mm/(m∙s^−2^)	RMS			The Maximum Value		
	Without Control	With Control	Control Effect	Without Control	With Control	Control Effect
End displacement	1.70	0.33	80.27%	3.56	1.33	62.73%
Middle acceleration	1.43	0.96	33.01%	5.19	2.38	54.19%

**Table 4:** The statistics data under random signal tests.

mm/(m∙s^−2^)	RMS			The Maximum Value		
	Without Control	With Control	Control Effect	Without Control	With Control	Control Effect
End displacement	1.14	0.23	79.23%	3.02	0.82	72.83%
Middle acceleration	0.97	0.67	30.32%	4.99	2.46	50.67%

## Data Availability

Data are contained within the article.
